# Functional Regulation of the Plasma Protein Histidine-Rich Glycoprotein by Zn^2+^ in Settings of Tissue Injury

**DOI:** 10.3390/biom7010022

**Published:** 2017-03-02

**Authors:** Kristin M. Priebatsch, Marc Kvansakul, Ivan K. H. Poon, Mark D. Hulett

**Affiliations:** Department of Biochemistry and Genetics, La Trobe Institute for Molecular Science, La Trobe University, Melbourne, VIC 3086, Australia; kmpriebatsch@students.latrobe.edu.au (K.M.P.); m.kvansakul@latrobe.edu.au (M.K.)

**Keywords:** histidine-rich glycoprotein, plasma protein, zinc, tissue injury, wound healing, cancer

## Abstract

Divalent metal ions are essential nutrients for all living organisms and are commonly protein-bound where they perform important roles in protein structure and function. This regulatory control from metals is observed in the relatively abundant plasma protein histidine-rich glycoprotein (HRG), which displays preferential binding to the second most abundant transition element in human systems, Zinc (Zn^2+^). HRG has been proposed to interact with a large number of protein ligands and has been implicated in the regulation of various physiological and pathological processes including the formation of immune complexes, apoptotic/necrotic and pathogen clearance, cell adhesion, antimicrobial activity, angiogenesis, coagulation and fibrinolysis. Interestingly, these processes are often associated with sites of tissue injury or tumour growth, where the concentration and distribution of Zn^2+^ is known to vary. Changes in Zn^2+^ levels have been shown to modify HRG function by altering its affinity for certain ligands and/or providing protection against proteolytic disassembly by serine proteases. This review focuses on the molecular interplay between HRG and Zn^2+^, and how Zn^2+^ binding modifies HRG-ligand interactions to regulate function in different settings of tissue injury.

## 1. Introduction 

**Figure 1:**
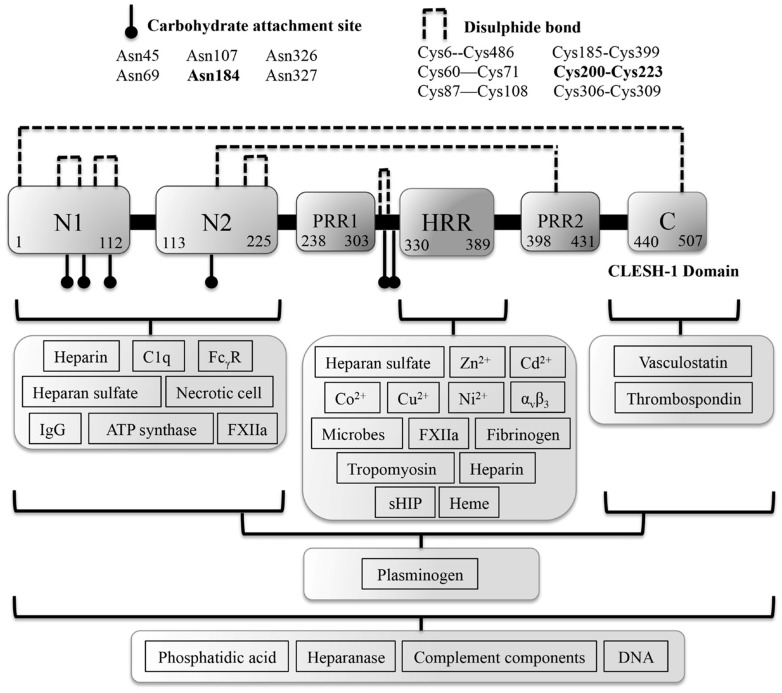
Domain structure and ligands of histidine-rich glycoprotein (HRG). Proposed domains of human HRG are shown with ligands listed for each domain/region based on current literature [3,13,14,15]. The predicted structure of HRG consists of two N-terminal cystatin homology domains (N1 and N2), a histidine-rich region (HRR) flanked by two proline-rich regions (PRR1 and PRR2) and a C-terminal domain (C). N-linked glycosylation sites and disulphide bonds are indicated in bold. The only HRG domain of known structure is the recently solved N-terminal cystatin domain 2 (N2) from rabbit, which shares 80% sequence identity with its human counterpart [16]. Figure adapted from Poon et al. [3]. IgG: Immunoglobulin G; sHIP: streptococcal histidine-rich glycoprotein interaction protein.

It was almost half a century ago that the histidine-rich glycoprotein (HRG) was first described from human serum, and since then there have been significant advances in understanding its functional role in a range of physiological and pathological settings [1,2,3,4]. In particular, the regulation of HRG function upon binding of Zn^2+^ or changes in environmental pH has become a recurring observation in recent years. This metal and pH-sensing feature of HRG can be attributed to the unusually high content of histidine residues that are localized as tandem repeats motifs, in a histidine-rich region (HRR). The HRR enables HRG to alter its charged state through either Zn^2+^ binding or protonation of the histidine residues under acidic conditions, which in many cases appears to be pivotal for ligand interaction and impacts ligand affinity [4,5,6]. The importance of this Zn^2+^-sensing region is underscored by its evolutionary conservation within the kininogen family members of the cystatin superfamily, with the majority of the family members harboring a HRR or HRR-like sequence [6,7]. In addition to the HRR, HRG is comprised of several other domains, including two cystatin N-terminal homology domains (N1, N2), two proline-rich regions (PRR1, PRR2) that flank the HRR, and a C-terminal domain (Figure 1) (for HRG gene and structural homology refer to Jones et al. [4]) [4,5,7,8]. Given the multi-domain character of HRG that mediates interaction with a large array of different ligands, the substantial number of biological processes in which this protein is implicated is not surprising. Therefore, the focus of this review will be the interplay between the Zn^2+^ and pH in regulating HRG immune and vascular processes that occur at sites associated with tissue injury or tumour growth [3,6,9,10,11,12].

The resolution of tissue injury involves specific communication between cells at the wound bed (e.g., epithelial cells, mesenchymal cells and leukocytes), in order to enable tissue repair through promoting cell proliferation [17]. Although these events may share common processes in the mechanisms that underpin acute or chronic wounds and tumourigenesis, there are clear differences. Wound-healing mechanisms culminate at the damaged epithelium and follow a normal wound-healing response, while tumours mimic that of chronic wounds and take advantage of these dysregulated mechanisms to move beyond tissue borders [17,18,19]. Therefore, considering HRG is constitutively expressed from liver parenchymal cells and maintained at a plasma concentration of 100–150 µg/mL [1,2], it will be rapidly available at the wound site when inflammatory events begin the formation of the hemostatic plug through platelet adherence, in order to provide a temporary extracellular matrix (ECM) scaffold for cell migratory processes [20]. In addition, these processes also generate the necessary environment for the release of Zn^2+^ through platelet degranulation and mechanical cell stress that in combination can elevate the serum concentrations above normal homeostatic values of free Zn^2+^ from 0.2–2.0 μM to 19–50 μM [4,21,22,23,24,25,26,27]. This supports the growing evidence that a large proportion of the aforementioned immunological and vascular functions of HRG are dependent on the presence of Zn^2+^, with the pH potentially providing an additional layer of control by regulating metal coordination and overall net-charge. Furthermore, pH and Zn^2+^ are capable of regulating the proteolytic disassembly of HRG individual domains from serine proteases such as plasmin [28]. These mechanisms provide HRG with the ability to regulate its function by sensing changes in pH and Zn^2+^ levels at sites of injury. Furthermore, as these factors can also vary depending on the nature of the injurious site, such as an acute or chronic wound, or at a site of tumour growth, this suggests that the regulation of HRG function will be specific within different sites [6,10,11,26].

## 2. The Functional Significance of Zn^2+^ Coordination Geometry in Histidine-Rich Glycoprotein Function

**Figure 2:**
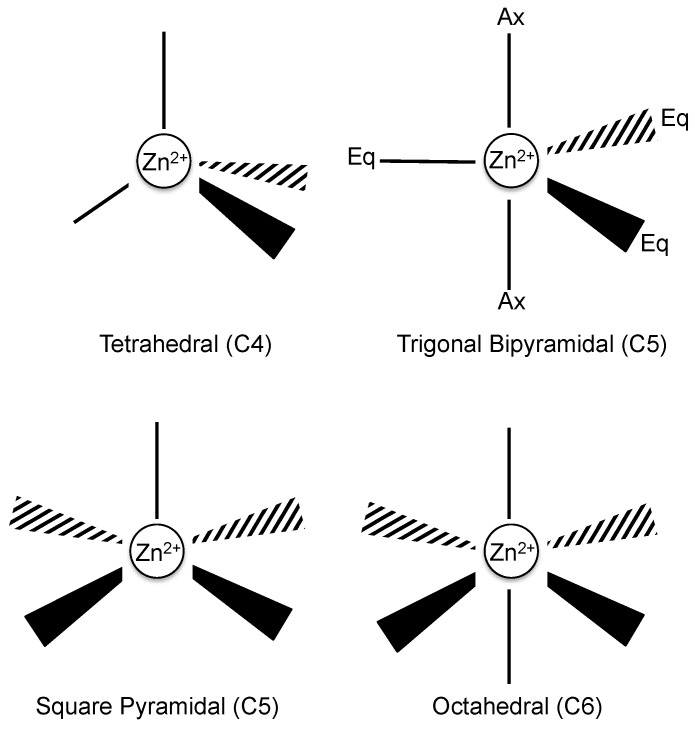
Ideal Zn^2+^ coordination geometries. The figure represents the types of ideal Zn^2+^ coordination geometries such as tetra (C4), penta (C5) and hexa (C6). The Ax = axial and Eq = equatorial define the ligand donor that participates in accommodating the Zn^2+^-binding site complex. Figure adapted by Patel et al. [29].

Zinc is the second most abundant transition element in the human body and acts as a cofactor to provide structural stability as well as other regulatory roles to proteins [29]. Examples of these regulatory roles extend to Zn^2+^ binding sites that utilize alternate combinations of ligand donors via multiple Zn^2+^ coordination geometries, to enable the regulation of redox reactions, environmental Zn^2+^ availability, catalytic activity, oligomeric states of proteins and altering the structural conformations of protein domains to bind DNA, RNA, lipid and protein ligands [3,4,30]. Structural data indicates that there is a trend in the preferred type of ligand geometry for Zn^2+^ binding, based on whether a Zn^2+^ binding site mediates either a structural or catalytic function. In general, Zn^2+^ can be configured to three types of coordination complexes that include tetra (C4), penta (C5) and hexa (C6), which are defined based on the number of ligand donors that participate in a Zn^2+^ binding site complex (Figure 2) [30]. The common amino acid side chains that participate in Zn^2+^ tetrahedral geometry include that of histidine (via nitrogen), glutamic or aspartic acids (via oxygen) and cysteine (via sulfur), with each side chain capable of engaging with one or two Zn^2+^ ions [29]. It should also be noted that in some cases the amino terminus of a metalloprotein has been observed as a metal-binding ligand [31,32,33].

The tetrahedral geometry is the most prevalent of Zn^2+^ coordination complexes in biological systems [29]. However, there are slight shifts in geometry depending on the use of Zn^2+^ binding sites for structural or catalytic roles. Structural roles for C4 geometries dominate over catalytic roles, with the latter showing an increase in C5 and C6 coordination complexes [29]. The different usage patterns of certain Zn^2+^-binding geometries may provide functional insight; however, there are cases where the ligand configurations in the Zn^2+^-binding pocket may not indicate its biological function. In some cases, more than one biological role can be attributed to the same Zn^2+^-binding pocket configuration, as is observed for ZnS_4_ arrangement [30]. This ZnS4 arrangement has been attributed with modulating a number of different functions, such as (i) enabling dimer formation (protein-protein interactions) in alcohol dehydrogenase and nitric oxide synthase [30]; (ii) acting as a redox switch in the Heat shock protein 33 (Hsp33) protein [30]; and (iii) facilitating the activation of ligands for downstream chemical reactions, as observed for the Ada protein [30]. Furthermore, Zn^2+^-binding sites localized around the flexible loop regions of proteins tend to show plasticity, whereby the ligand configurations alter each time the Zn^2+^ ions associate and dissociate from the binding site [30,34]. This highlights how different combinations of ligand donors in the Zn^2+^ tetrahedral geometry can subtly influence and sometimes dictate the functional roles of proteins.

A model of Zn^2+^-binding domains of HRG has been proposed that involves the tandem histidine-rich repeats (GHHPH-like motifs) from the HRR, forming a twisted elongated helical core that projects outwards the histidine side chains, with the ligand donors being provided through the nitrogen of histidine [4]. The Zn^2+^-binding pattern of this tandem repeat is unusual, due to the presence of proline, as this amino acid is not represented in the top 75% of structural or 46% of catalytic Zn^2+^-binding domain sites. It is proposed that the presence of proline mediates the predicted helical twist formation of the HRR, promoting histidine residues of the GHHPH repeat motifs to protrude outwards, enabling the fold to accommodate Zn^2+^ binding [4,29]. Also, as mentioned previously, the types of ligand donors involved in the coordination geometry of divalent metals may aid in identifying the functional roles of proteins. Therefore, it is tempting to speculate that examining the functional role of proteins that utilize similar ligand donors to accommodate Zn^2+^ may also provide insight into HRG activities. In particular, the ZnN_3_ configuration (where N_3_ indicates the involvement of three histidines) has been shown to correlate with catalytic or inhibitory roles, intracellular transport of Zn^2+^ and structural stability of proteins [30].

Given there is no evidence to support catalytic (C5 or C6 geometries) or Zn^2+^ transporter activities for HRG, in the context of wound healing, the Zn^2+^ may play a structural role (C4 geometry) for HRG by promoting protein stability or possibly acting as a cofactor between HRG-HRG interactions to form higher-order oligomeric states [3]. Another possibility is that by protonating the histidines through an acidic environment generated during inflammation, or compensating for the loss of charge by binding Zn^2+^ during the re-epithelialization of acute wound healing, may provide ionic repulsion against serine proteases such as plasmin that would otherwise proteolytically alter HRG structure and function [6,9,28,30,35]. However, fluctuations in pH and Zn^2+^ levels differ substantially in acute and chronic wounds, with chronic wound pH returning to physiological levels directly after the acute phase [6]. Therefore, the nature of the wound-healing process (i.e., acute or chronic) may differentially regulate the ability of HRG to coordinate with Zn^2+^.

### 2.1. HRG Binding to Other Divalent Metal Cations

The levels of metals in tissues or circulating throughout the plasma of individuals are tightly regulated and if perturbed can contribute to the onset of disease [26]. In particular during tissue repair it has been shown that Zn^2+^ and Ca^2+^ levels are elevated early in acute wounds [9,11], while aberrant levels of other divalent cations have been shown to occur in tissues that are compromised by cancer progression [26].

The majority of metal interactions attributed to HRG (in addition to Zn^2+^) were initially identified using rabbit HRG (rbHRG). The earliest studies that identified other divalent cations binding by HRG were performed by Morgan [13], who investigated the ability of divalent cations to displace heme from the HRR [13]. This indirect assay demonstrated that in HRG, Cu^2+^ (almost completely), Zn^2+^ (50%), Ni^2+^ (50%), Cd^2+^ (30%) and Co^2+^ (10%) were able to bind and displace heme from HRG, with further studies adding mercury (Hg^2+^) to the list from rbHRG [13,36]. These interactions of HRG with Cu^2+^ and Zn^2+^ were confirmed further using synthetic peptides (GHHPH) of the HRG HRR [37]. Interestingly, this study described unpublished results suggesting that these immobilized fragments when bound either to Cu^2+^ or Zn^2+^, showed altered selectivity patterns in pull^-^down assays from human plasma [37]. Other reported cases of histidine-rich sequences or flexible loop regions that have been described to interact with divalent metals to regulate a number of functions, include (i) transporter properties by TjZNT1 [38] and ZnuA [39]; (ii) storage capabilities by the cytoplasmic protein Hpn [40]; (iii) initiate catalysis of the superoxide anion into oxygen and hydrogen peroxide by the Cu, Zn superoxide dismutase [41] and (iv) enable the formation of plaque-thread junctions by the *Mytilus californianus* foot protein 4 (mcfp-4) protein [42]. This proposes that the binding of metals by HRG will be dictated by their distribution in tissues, something that will vary depending on the particular physiological or pathological setting, that in turn will regulate ligand binding and therefore function of HRG.

The amino acid identity shared between HRG and rbHRG is 65.3%, with sequence conservation highest for the N- and C-termini. The central HRR of HRG (GHHPH-like motifs) and rbHRG (GHHPH/GHPPH/GPPPH-like motifs) contain a variable number of histidine-rich tandem repeats, with the HRR slightly longer for the rbHRG [43,44]. Therefore, the affinity and stoichiometry calculated for these divalent cations for rbHRG would appear likely to differ for HRG [29,43]. Numerous studies have confirmed that rbHRG binds a number of different metals such as Cu^2+^, Zn^2+^, heme, Hg^2+^, Ni^2+^, Cd^2+^ and Co^2+^. The affinity of rbHRG for these metals follows the universal order of affinity of the Irving-William series, with affinities near the dissociation constant (K_d_) value of 1 μM and stoichiometries of ~10 for almost all these metals [36,45,46]. Interestingly, the number of divalent metal-binding sites in rbHRG were shown to be consistent for Zn^2+^ binding by HRG, which could be considered surprising given that HRG contains slightly more histidines in the HRR to act as ligand donors and hence accommodate more Zn^2+^ binding sites. Furthermore these additional histidines may contribute to the almost two-fold greater affinity for Zn^2+^ of HRG (K_d_: 12.41 μM) over rbHRG (K_d_: 22.78 μM) as observed by Kassaar et al. [47].

Recently, the affinities for Zn^2+^ (K_d_: 2.85 μM) > Ni^2+^ (K_d_: 3.35 μM) > Cu^2+^ (K_d_: 3.72 μM) > Co^2+^ (K_d_: 9.39 μM) and stoichiometries for Zn^2+^ (9–10 sites), Ni^2+^ (13 sites), Cu^2+^ (6 sites) Co^2+^ (13 sites) of HRG were defined by isothermal titration calorimetry (ITC) [48]. The number of sites for Zn^2+^ was consistent with Kassaar et al. [47], although data by Priebatsch et al. [48] demonstrated greater affinity for Zn^2+^ (K_d_: 2.85). The discrepancy between the observed affinities was proposed by Priebatsch et al. [48] to be potentially due to the non-favorable reaction conditions used in Kassaar et al. [47]. The comparison between the orders of affinity for rbHRG (Cu^2+^: K_d_: 0.2 μM > Zn^2+^ K_d_: 1 μM > Ni^2+^ K_d_: 1.3 μM > Co^2+^: K_d_: 2.1 μM) by Morgan et al. [36] and for HRG (Zn^2+^: Kd: 2.85 μM > Ni^2+^: K_d_: 3.35 μM > Cu^2+^: K_d_: 3.72 μM > Co^2+^: K_d_: 9.39 μM) by Priebatsch et al. [48], demonstrates considerable differences. A rationale as to why HRG does not conform to the universal order of affinity of the Irving-William and exhibits the greatest affinity for Zn^2+^, compared to rbHRG exhibiting the greatest affinity for Cu^2+^, may be attributable to the nature of the histidine-rich tandem repeats. The HRR of HRG consists of a GHHPH-like motif (of which 11 are found in human) and contains a greater concentration of histidines compared to the rbHRG HRR (GHHPH/GHPPH/GPPPH-like motifs) [43,44]. Furthermore, the only available ligand donor in the GHHPH-like motif capable of coordinating with Zn^2+^ is the nitrogen from histidines, which subsequently also exhibits preference to Zn^2+^ ions over other divalent metals and therefore suggests Zn^2+^ ions may favor the richest histidine sequences [29,46]. Lastly, the difference in divalent metal affinities and stoichiometries between HRG and rbHRG are also likely to also extend to the differences in the histidine-rich tandem repeats, coordination geometries (Refer to Section 2) or possibly the type of metal-binding experiments undertaken, as Morgan [36] used absorbance titrations and Priebatsch et al. [48] used ITC.

**Figure 3:**
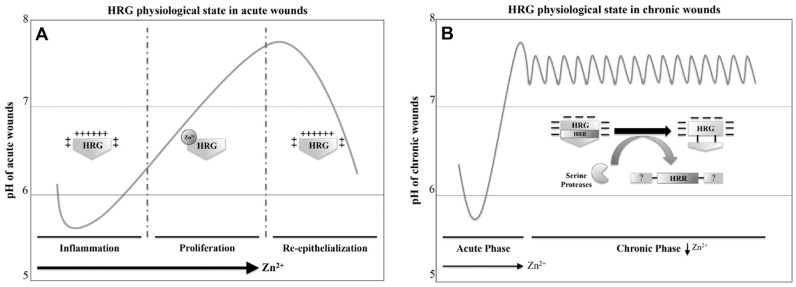
Potential physiological representation of HRG during the different stages of acute or chronic wounds. HRG is exquisitely sensitive to changes in environmental pH, given the charged state of the histidines controlling its ability to coordinate with Zn^2+^ and thus regulating its previously mentioned activities such as its antimicrobial properties [35,49]. In addition, the release of peptide fragments containing the HRR are unlikely to contribute to its antimicrobial or anti-angiogenic activities in acute wounds, as there appears to be proteolytic protection of HRG during all stages of acute wound healing, possibly due to the positive charge generated during the inflammation and re-epithelialization stage or Zn^2+^ elevation during the proliferation stage (Figure 3A) [6,9,11,12]. Therefore the exact role of fragments released in chronic wounds remains unclear, as the alkali and hypozincemia environment does not favor the required charge associated with antimicrobial activity or Zn^2+^ coordination with its anti-angiogenic peptide [6,11,12,35,49]. In addition to plasmin and elastase, other proteases such as matrix metalloproteinases (MMP) -8, MMP-9, cathepsin G and urokinase-type plasminogen activator (uPA) have shown harbor-elevated activity in chronic wounds and therefore could be prime candidates for the proteolytic breakdown of HRG observed in the chronic wound fluid fraction [35,50]. Interestingly, the study looked at the activity of these proteases over a pH range and observed optimum activity for most between pH 7–9, with in vivo data from 43 patients’ chronic wound fluid (derived from pressure, leg and diabetic foot ulcers) demonstrating that elastase is the most predominant protease contributing to impaired wound healing [50]. These pH values corroborate with the pH values observed in chronic wounds and therefore reinforce the possibility that proteases that act on HRG are under chronic wound settings (Figure 3B) [6,35]. This suggests HRG function can be regulated throughout the different phases of the acute or chronic wound-healing process. These pH curves in the figures were adapted from Schneider et al. [6].

Interestingly, fragments of the HRG HRR and native rbHRG exhibited altered Zn^2+^ binding under low pH, with 50% of Zn^2+^ displaced at pH 6.0 and almost complete dissociation (<10% remaining) at pH 5.5 [36,37]. Considering evidence for the importance of Zn^2+^ levels in a tissue injury setting, it is highly likely that HRG is associating with Zn^2+^ during the transition into the proliferative stage as physiological pH normalizes, from the acidic environment during the inflammatory or re-epithelialization stage of acute wound healing (Figure 3A) [6]. This suggests that the pH is also acting as a regulator of HRG HRR by controlling the binding of metals and ligands.

## 3. HRG Binds Activated Factor XIIa, Plasminogen, Heparin, Fibrinogen and Fibrin to Regulate Coagulation

During the formation of blood clots at sites of tissue injury, the ruptured vessel wall (intrinsic pathway) exposes connective tissue to enable platelet and collagen binding in order to form the molecular scaffold between the damaged vessel wall and platelets [14,18]. The cell surface and secretory vesicles from platelets provide many of the necessary coagulation factors that ultimately enable the activation of the serine protease thrombin. The production of thrombin permits the proteolytic disassembly of fibrinogen into fibrin fragments to facilitate its reassembly into thin filaments that weave and interlace around the platelet plug to provide structural stability [18,51]. During thrombus development, HRG has been shown to enhance coagulation and fibrinolytic events in in vivo models using HRG-deficient mice [52]. The molecules involved in these processes that HRG has been proposed to bind and regulate are factor XIIa (FXIIa), plasminogen (discussed in Section 5), heparin, fibrinogen (and its cleaved form fibrin). Importantly, Zn^2+^ has been shown to regulate most of these interactions [14,28,47,53,54].

### 3.1. HRG Serves as a Pro-Coagulant Molecule by Neutralizing Heparin

The ability for HRG to bind heparin is well documented, with early studies characterizing its heparin-binding domains (N1N2, HRR) and capacity to inhibit the anticoagulant properties of heparin in plasma ex vivo, but only in the presence of Zn^2+^ [55,56]. Recently, the biophysical characterization of the interaction between HRG and heparin was investigated by Kassaar et al. [47]. This study used ITC to demonstrate that supplementing HRG with increasing concentrations of Zn^2+^ (1 μM and 5 μM) altered the binding mechanism between HRG and unfractionated heparin, even though the affinities in the absence and presence of Zn^2+^ (1 μM) appeared to remain the same. Collectively, these data suggested that the interactions(s) between HRG and heparin occur by different modes and are modulated by Zn^2+^. However, it was noted that the affinities of HRG for unfractionated heparin in the presence of Zn^2+^ are likely greater, given ITC was unable to quantify the Zn^2+^-dependent mode of binding when supplemented with 5 μM of Zn^2+^ [47].

In an attempt to better quantify the multiple binding enthalpies occurring between HRG and a heterogeneous population of heparin (unfractionated) by ITC, the study by Kassaar et al. [47] established an ELISA protocol. Interestingly, the reported affinities in the absence of Zn^2+^ were significantly greater as measured by ELISA (K_d_: 32.9 nM) than by ITC (K_d_: 0.41 μM), which was speculated to involve the Zn^2+^-dependent mode of binding that could not be quantified by ITC. The addition of Zn^2+^ (1 μM) to HRG enhanced affinity for (unfractionated) heparin (5.1 nM), similar to previous reports [14,47]. However, when low molecular weight heparin (LMWH – 6850 Da) was used in the absence and presence of Zn^2+^ (1 μM), the affinities remained similar. This inferred that between LMWH (6850 Da) and unfractionated heparin (3 kDa–30 kDa), Zn^2+^ enhances HRG affinity for larger heparin chain lengths. Lastly, in regards to stoichiometries, the data support HRG N1N2 domain binding at a range of heparin chain lengths, with lower concentrations of Zn^2+^ (1 μM) enabling up to two molecules of HRG to interact with a single heparin molecule and higher concentrations of Zn^2+^ (5 μM) averting the 2:1 HRG-heparin complex.

This leaves the exact binding mechanism between HRG and heparin at higher Zn^2+^ concentrations unclear., although it was inferred that increasing the concentration of Zn^2+^ might also increase the binding capacity of the HRR and enable a greater number of heparin molecules to associate per HRG molecule. This is a possible scenario, given the above experiments were performed in the presence of 1 μM Zn^2+^, which is 100 times less than required to fully saturate all 10 Zn^2+^ binding sites of HRG. The emphasis on the lower concentration of Zn^2+^ was due to the observation that the 2:1 complex (HRG/heparin) was averted when Zn^2+^ was supplemented five-fold (5 μM), reiterating that the regulatory mechanism by Zn^2+^ of the HRG-heparin interaction requires further investigation [47].

### 3.2. HRG Binds Activated Factor XIIa to Subdue Its Pro-Coagulant Activities

HRG has been proposed to regulate the coagulation system, by engaging FXIIa, as no binding was observed for XII, XI or XIa [14]. The HRG-FXIIa complex has been shown to attenuate clot formation. The role for HRG during clotting was further supported when comparing plasma that has been immunodepleted and subsequently supplemented with HRG, with similar results obtained in HRG-deficient mice [14,52]. Interestingly, the affinity of HRG for FXIIa increases in a Zn^2+^-dependent manner that could potentially impact clotting. However, even though the presence of Zn^2+^ increased the affinity of HRG for XIIa by 1000-fold (K_d_: 9 pM), the interaction was still observed in the absence of Zn^2+^ at physiological pH (K_d_: 1.6 nM). Significantly, the affinity of HRG for FXIIa is the highest described for any HRG ligand. The role of Zn^2+^ in this setting was postulated to be an additional control element for HRG to regulate FXIIa departure from the thrombus site or modulate further FXIIa activation at the fibrin clot [14,28]. It is also worth noting that the presence of DNA and RNA released from apoptotic and necrotic cells, or the release of polyphosphates from activated platelets, can facilitate the intrinsic pathway through FXIIa activation. Therefore, given that HRG can bind the phosphate head group of phosphatidic acid, it is no surprise that HRG can bind the phosphate-backbone of nucleic acid and hence reduce the pro-coagulant properties of DNA and RNA [15,57,58].

### 3.3. HRG Interacts with Fibrin(ogen) to Regulate the Formation of the Platelet Plug

HRG has also been shown to bind the zymogen precursor fibrinogen and its proteolytic product fibrin, with initial studies suggesting that the observed prolonged thrombin time can be attributed to the HRG-fibrinogen complex hindering fibrin turnover [53]. However, recent evidence has suggested that this was due to HRG inhibiting assembly of the fibrin monomers, as HRG bound preferentially to fibrinogen in the presence of Zn^2+^ and did not impede thrombin generation of fibrinopeptides A and B that assemble the fibrin meshwork [59,60]. This leaves the mechanism of the HRG-fibrinogen complex formation unclear, as both reports of binding demonstrate the presence of divalent cations as crucial, with Ca^2+^ and Zn^2+^ shown to be candidates. Furthermore, the presence of ethylenediaminetetraacetic acid (EDTA) prevented HRG association with fibrinogen and within the fibrin clot, with increasing Ca^2+^ levels restoring these interactions [53,60]. In the Zn^2+^-dependent case, binding was identified between fibrinogen and fragments of HRG derived from the HRR [60]. It should be noted that the type of divalent cation might influence specificity and regulatory function of HRG with fibrinogen, something that requires further investigation.

To clear up the conflicting findings of the previously described HRG and fibrin(ogen) complexes, a study by Vu et al. [61] examined the interaction by surface plasmon resonance (SPR) in the presence of either Ca^2+^ or Zn^2+^. The experiment demonstrated that the interaction of HRG with fibrin(ogen) was Zn^2+^ dependent and that this complex could be observed in vivo (plasma) [61]. It is worth mentioning that fibrin(ogen) can also bind Zn^2+^; it was therefore postulated that Zn^2+^ may be acting as a cofactor that enables the HRG-fibrin(ogen) complex to form simultaneously [61]. The rationale behind the earlier conclusions involving Ca^2+^ suggested that the purification procedure used to isolate HRG from plasma had sites preoccupied by Zn^2+^ [53,61]. This reiterates the importance of the purification procedure used to isolate HRG from plasma, prior to investigating HRG functional properties [15,61]. The study by Vu et al. [61] then examined the role of HRG in the HRG-fibrin(ogen) complex, since the HRG interaction with fibrinogen did not prevent the conversion of fibrinogen to fibrin by thrombin [61]. The major findings showed that HRG avidly competes with thrombin and factor FXIIa for fibrin. Therefore, once the fibrin matrix assembles, it was suggested that displacing either thrombin or FXIIa from fibrin by HRG provides HRG with antithrombotic and pro-fibrinolytic properties [61]. This in combination with previous reports—including HRG-fibrin complex sheathing the fibrin clot to enable bacterial entrapment/killing of microbes, tethering immune cells and sequestering hemostatic factors (FXIIa, heparin and plasminogen)—strongly suggests that HRG alongside Zn^2+^ plays a pivotal role in regulating hemostasis [14,28,35,49,52,54,61,62,63].

### 3.4. A Summary of HRG Activities in Context of Hemostasis

Based on these observations, if one considers the role of HRG in coagulation in the context of pH at the cutaneous wound, the acidic environment during inflammatory events would not be expected to favour Zn^2+^ binding to HRG (Figure 3A) [5,6,36,37]. Therefore, the acidic pH may be negatively regulating the formation of HRG anticoagulant complexes involving HRG-FXIIa, HRG-DNA/RNA and HRG-fibrin(ogen), considering these interactions were observed either under physiological pH (pH 7.4) or in combination with Zn^2+^ [14,58,61]. The acidic environment would persist until the transition into the proliferative stage of wound healing where the physiological pH levels normalize to enable HRG to coordinate with Zn^2+^ and subsequently promote the aforementioned HRG-ligand complexes [6,36,37]. This combination of pH and Zn^2+^ may be integral in regulating HRG role in hemostasis, by ensuring HRG anticoagulant properties only coming into effect after the hemostatic plug is established.

As for HRG pro-coagulant properties, the neutralization of heparin appears more difficult to explain in the context of wound healing. The optimal environmental condition that expedites HRG association with heparin is in combination with Zn^2+^; however, HRG can still bind heparin under physiological pH (7.4) in the absence of Zn^2+^ and likely under acidic pH (5.5) based on heparan sulfate (HS) binding studies [47,56,64,65]. Therefore, HRG can constantly sequester heparin and potentially prevent heparin anticoagulant properties from coming into effect. The potential mechanism behind this observation is possibly explained by HRG ability to switch specificity between different lengths of heparin based on the environmental condition. In combination with HS binding studies, under physiological pH (7.4) the N1N2 domain is capable of associating with heparin of all sizes, while the HRR upon meeting the prerequisites of being Zn^2+^ bound or positively charged by acidic pH can bind heparin lengths of octasaccharide or greater, with maximum affinity to dodecasaccharides [47,56,64]. Therefore, considering antithrombin utilizes heparin chain lengths of octasaccharides for activation, HRG may be regulating antithrombin activation by switching specificity between heparin lengths of octasaccharide or greater [47,56,64,66]. Overall, these studies demonstrate HRG with both pro- and anticoagulant properties that appear to be regulated by pH and Zn^2+^.

## 4. The Antibacterial and Antifungal Properties of HRG at Sites of Infection

Antimicrobial peptides (AMPs) play a key role in innate immune defence against bacterial invasion. Besides aiding the elimination of bacterial pathogens, AMP have also been reported to have other biological properties ranging from the regulation of angiogenesis to assisting professional phagocyte recruitment (chemotaxis) [49]. The molecular signature of certain AMP peptides is the presence of a canonical heparin-binding domain sequence, defined as the Cardin (AKKARA) or Weintraub (ARKKAAKA) sequence [67]. The investigation of similar sequences in the N1N2 and HRR of the HRG suggested that these HRG domains might possess antimicrobial properties, as was confirmed by Kacprzyk et al. [67] using peptides derived only from the HRR. This antimicrobial activity was further investigated by Rydengård et al. [35,49] who examined the effect acidic pH and Zn^2+^ had on the HRG ability to potentiate the lysis of microbes (discussed in detail below), such as bacteria and fungi [49,67].

### 4.1. HRG Central HRR Core Contributes to Multiple Modes of Killing Bacteria and Involves Changes in pH

#### 4.1.1. HRG Antibacterial Properties under Acidic Conditions

The skin provides an important barrier against the constant assault from environmental, commensal and opportunistic microorganisms that otherwise may penetrate, thrive and infect the underlying tissue to cause further harm to the host [68]. In addition to this barrier function, the skin harbors antimicrobial attributes, such as an acidic exterior that has been described as the skin acid mantle. The mantle provides the necessary acidic environment to counter the natural flora of bacteria that resides at the skin surface and also works concomitantly with antimicrobial peptides to facilitate their protonation and hence activity in directing microbial clearance [68].

The environmental pH levels fluctuate in the inflammatory stage of wound healing and can drop up to 1.5 pH units [6]. This would leave local pH levels below the pK_a_ of histidine and alter HRG net charge to positive, leading to enhanced bacterial killing [49]. Therefore, it is tempting to speculate that this fluctuation in pH could promote HRG antibacterial activity during the inflammatory or re-epithelialization stage and Zn^2+^-dependent killing in the proliferative stage of acute wound healing (Figure 3A). This net-charge-related mechanism of bacterial clearance was observed under atomic force microscopy with clear disruption of the bacterial cell wall [49]. The antibacterial activity in relation to the net positive charge of HRG was attributed to the phospholipid constituents of the bacteria cell wall, with gram-negative killing proficiency greater than gram-positive bacteria due to the presence of stronger anionic lipids such as phosphatidyl-glycerol, phosphatidylserine and cardiolipin. Although these negative-charged phospholipids are located on the inner leaflet, it still gives an overall net negative charge to the cell wall and therefore was inferred as potential targets for AMPs such as peptides derived from HRG HRR [49,67].

#### 4.1.2. HRG Coordinates with Zn^2+^ Around Physiological pH to Maintain Antibacterial Activity

Similar to these antimicrobial mechanisms observed under acidic pH, HRG displays enhanced antibacterial activities against gram-negative and positive bacteria at physiological pH (7.4) in the presence of Zn^2+^ (50 μM). The pH and Zn^2+^-sensing property of HRG are regulated through the pK_a_ of the histidines (6.45): if the pH declines below this value, the histidines are protonated, whereas values above this would allow co-ordination with Zn^2+^, therefore generating the required positive charge for antibacterial activity under physiological and pathological conditions [49,67]. An example where the release of Zn^2+^ in a physiological setting may potentially facilitate the antibacterial properties of HRG has been observed at sites pertaining to tissue injury. Upon vasculature damage, the exposed substratum allows the recruited platelets to adhere and initiate downstream coagulation cascades through the release of effector molecules from granules, a process required to prevent further hemorrhaging by forming the fibrin clot. In addition to this well-established primary role, the localized distribution of Zn^2+^ from platelets (Discussed Section 1. Introduction) would correlate with enhanced bacterial clearance at sites of fibrin clots when HRG is present [24,49,53,67,69].

#### 4.1.3. HRG Central HRR Core is an Obstacle for Invading Microbes

The significance of HRG central histidine-rich core is underscored by invading pathogens having to counter its antibacterial properties in order to successfully infect the host. An example of invading pathogens having to counter the HRR antibacterial properties was observed by Streicher et al. [70], with the discovery of a virulence factor secreted from the AP1 strain of *Streptococcus pyogenes* (*S. pyogenes*) [70]. This virulence factor was titled streptococcal histidine-rich glycoprotein interaction protein (sHIP) (UniProtKB ID Q99XU0), due to its capacity to specifically target HRG and block the activities of the HRR. In addition, peptides derived from the HRR (GHHPH-like motif) were also ineffective in attenuating growth of *S. pyogenes* in medium, which demonstrates that even if peptides derived from the HRR can be proteolytically generated and released from the parent protein, the *S. pyogenes* will still thrive. Interestingly, sHIP ability to bind and act as a soluble decoy receptor for HRG remained constant even under acidic conditions [70].

However, despite how effective sHIP is against negating HRG antibacterial properties, there are earlier reports on HRG killing the same strain of *S. pyogenes* (AP1 strain) in the presence of Zn^2+^ and under acidic conditions. Primarily using mouse models, Shannon et al. [63] showed that HRG interacts with fibrin filaments at the wound site in order to capture and prevent the escape of invading bacteria systemically. Notably, this interaction of HRG with fibrin is Zn^2+^-dependent, which acts as the cofactor between these binding partners [27,61,63]. In addition, an infection model of *S. pyogenes* (AP1 strain) in HRG^-/-^ mice resulted in a marked reduction in survival rate, when compared to wild type. Interestingly, this survival rate could be restored to wild-type levels, once HRG was reconstituted into the plasma of *HRG*^-/-^ mice [63]. These models represent a strong case of what occurs in vivo, and supports the argument that HRG can prevent the growth and spread of *S. pyogenes* at the site of infection where the environmental milieu favors HRG antibacterial properties [63,70].

The ability of sHIP to counter somewhat both the acidic environment and antimicrobial peptides through secretion of the sHIP virulence factor, may explain why the AP1 strain is more pathogenic compared to the less virulent SF370 strain of *S. pyogenes* [70]. It is worth noting that HRG activity in the presence of Zn^2+^ was not directly studied and therefore may play an important role [70].

### 4.2. HRG Exhibits Antifungal Activity by Utilizing Its Histidine-Rich Core

In addition to antibacterial activities, the HRG HRR was also shown to harbor antifungal properties against a commensal eukaryote *Candida* that normally resides at the skin or mucosal surfaces [35,71]. *Candida* infections can be quite severe, with aggressive disease capable of mediating life-threatening sepsis [71]. The microenvironment plays an integral role in the antifungal properties of HRG and other AMPs against different *Candida* strains (*C. Parapsilosis*, *C. albicans*, *C. glabrata* and *C. Krusei*), with complete killing observed under acidic conditions (pH 5.5) in contrast to low levels of killing at physiological pH (pH 7.4). This illustrates the importance of the cationic charge of AMPs including HRG in the protection against *Candida*. Similar to the charged-based interaction induced through protonation of the HRR for antibacterial activity, the mechanism of HRG antifungal activity was shown to involve permeabilization of the fungal membrane leading to leakage of cytoplasmic contents [35,49]. Liposome-based assays identified ergosterol as the preferred HRG ligand at low pH (6.0), leading to liposome leakage, and considering the HRG antifungal activity is abolished in the presence of heparin, it is likely that this lipid interaction is mediated through the HRR [35]. Indeed, a scan using overlapping peptide fragments of HRG identified the HRR-PRR2 as exhibiting the most prominent antifungal activity. Furthermore, HRG lacking the HRR and C-terminal domain showed no activity even at low pH [35].

Whether individual domains of HRG are released that harbor antimicrobial properties following exposure to proteases at sites of tissue injury has not yet been defined. There has been only one reported study by Thulin et al. [12] that observed, in the serum of cancer patients, the proteolytic (protease remains unknown) release of individual domains from HRG that harbored a specific function. The individual domain was observed as a ~33 kDa complex by Western blot analysis under non-reducing conditions and found to contain a 25-amino acid anti-angiogenic peptide (termed HRGP330: HHSHEQHPHGHHPHAHHPHEHDTHR) [12,35,49]. Interestingly, work by Rydengård et al. [35] examined HRG in biological fluids that were taken from injured tissue; these included the plasma, serum, acute wound fluid, chronic wound fluid, platelets and the fibrin clot [35]. These samples were analysed by Western blot under both reducing or non-reducing conditions. Surprisingly, the detection of a partially HRG-degraded 36 kDa species using an HRR (GHHPH)-specific antibody in the chronic wound fluid fraction (derived from leg ulcers) under non-reducing conditions, was not discussed. However, the acute wound fluid fraction only contained intact HRG, even when analysed under reducing conditions. These data suggest that factors protecting HRG from proteolytic processing are not present during the stages of chronic wound healing [35]. In regards to what proteases may be acting on HRG in chronic wounds, there are reported cases of elevated levels of serine proteases such as elastase and cathepsin-G released from polymorphonuclear neutrophils that may be prime candidates [72]. Therefore, HRG may have the potential to be differentially regulated between acute or chronic wound-healing processes.

The study then investigated how HRG is distributed at the formation of the fibrin clot via using an ex vivo model involving human plasma and fluorescein isothiocyanate (FITC)-labeled HRG. The model clearly illustrates the distribution of HRG at the clot boundaries, therefore providing a sheath that was postulated to act as a barrier against bacterial or fungal invaders [35]. This observation was further supported in a mice knockout HRG^-/-^ model that showed enhanced fungal growth in the plasma and at the fibrin clot site compared to wild-type counterparts [35]. It should be mentioned that these studies did not examine HRG antifungal properties in the presence of Zn^2+^ [35]. Therefore, HRG might exhibit similar or enhanced antifungal properties in the presence of Zn^2+^, possibly to compensate for the loss in charge as the acute wound milieu becomes more alkaline during the course of tissue regeneration [6,35].

## 5. Plasmin-Mediated Cleavage of HRG Alters Its Biological Function and Regulates Plasminogen Activation 

The serine protease plasmin is the product of the plasminogen activation (PA) system that specifically targets substrates for proteolytic cleavage to mediate physiological processes involved in fibrinolysis, tissue remodeling and wound healing [73]. The key molecular components of the PA system that are required to proteolytically activate plasminogen to generate plasmin include urokinase type-PA (u-PA) and tissue-type-PA (t-PA). The u-PA resides at the cell surface whereas t-PA remains bound at sites of fibrin clots. Both proteases proteolytically convert plasminogen into plasmin, thus enabling the dissolution of the fibrin clot [73]. The binding of HRG to plasminogen has been shown to regulate plasmin generation through sequestering soluble plasminogen to glycosaminoglycan (GAG) surfaces and subsequently aid its activation by t-PA, with this sequestering potentiated in the presence of Zn^2+^ or low pH [54]. To date, only plasmin has been investigated in detail by Poon et al. [28], with the study demonstrating that HRG is a substrate for plasmin that modifies HRG multi-domain structure to regulate feedback mechanisms involved in the PA system and alter ligand affinity for HS, necrotic cells and plasminogen [28,74].

To understand the structural changes on HRG caused by plasmin cleavage, the study by Poon et al. [28] investigated plasmin-mediated cleavage in vitro by sodium dodecyl sulfate polyacrylamide gel electrophoresis (SDS-PAGE) analysis under reducing or non-reducing conditions, which indicated the formation of multiple fragments only under reducing conditions. Furthermore, the HRG fragments generated under reducing conditions could also be demonstrated ex vivo, when the plasma was supplemented with additional plasmin. To identify the fragments generated, Western blot and Edman degradation analysis revealed the release of the C-terminal domain initially, with the subsequent release of the N1N2, HRR-PRR2 and C-terminal domains when protease treatment was prolonged. Therefore, under non-reducing conditions, the HRR-PRR2 and C-terminal domain must remain attached to the N1N2 through inter-disulphide bonds.

Proteases other than plasmin have been proposed as potential candidates to mediate the release of active individual domains of HRG, including kallikrein and elastase; however, they are yet to be investigated in detail [28]. To date, the only individual domain of HRG that has been extensively studied is the HRR, with partial or full-length fragments used in multiple studies demonstrating anti-angiogenic, antimicrobial and endotoxin-neutralizing activity [49,67,75,76,77,78,79]. Poon et al. [28] further compared some of the known biological roles of HRG when subject to initial or prolonged exposure of HRG to plasmin. The initial cleavage of HRG by plasmin reduced its ability to bind HS, while enhancing its binding to plasminogen and necrotic cells. In contrast, prolonged exposure of HRG to plasmin restored its activities to that observed for intact HRG. The proposed biological significance of the initial plasmin cleavage of HRG may include (i) the reduction of HRG binding HS in order to promote the binding of other HS-binding proteins such as pro-angiogenic proteins fibroblast growth factor (FGF)-2 and heparanase (HPSE); and (ii) providing a negative feedback loop for the PA system, with plasmin-cleaved HRG acting as a soluble decoy receptor to compete with the intact HRG for plasminogen to regulate plasmin turnover [28].

It should be noted that plasmin-mediated cleavage of HRG is negatively regulated when Zn^2+^ is present or when physiological pH is acidic [28,80]. The exact mechanism behind acidic pH or presence of Zn^2+^ regulating HRG proteolysis remains unclear. However, the charge induced by protonation or binding of Zn^2+^ by the HRR may cause it to adopt a unique conformation that is proteolytic resistant. Alternatively, the acidic pH and/or Zn^2+^ may simply be regulating plasmin activity. The latter has been suggested, as a recent study demonstrates that Zn^2+^ is capable of interacting with plasmin, t-PA and u-PA to reduce their activities and subsequently attenuate clot dissolution [27]. In addition, an acidic milieu has been shown to almost completely inhibit plasmin activity [50]. This therefore suggests that the acidic wound milieu and the transition element Zn^2+^ inhibits plasmin activity, rather than a protective mechanism of HRG HRR becoming positively charged through Zn^2+^ coordination or protonation of the histidines via acidic pH [27,50].

Plasmin cleavage provides HRG with a unique regulatory mechanism, with cleavage by plasmin preventing the “normal” functions of intact HRG involving its modular domain binding of various ligands to mediate specific immune and vascular functions. Furthermore, plasmin-mediated cleavage of HRG may provide additional functions, as proteolytic disassembly could promote other independent functions of its domains [28]. Indeed, the release of individual domains may be a possibility, considering there is evidence to suggest that a glutathione adduct observed on the rbHRG N2 domain (80% sequence identify to human) may utilize a redox-mediated mechanism [16]. Although plasmin-mediated cleavage of HRG influences its ability to regulate the PA system, further investigation is required to determine if other proteolytic modifications of HRG take place by kallikrein or elastase [28].

Additionally, what is interesting to note is that the acidic pH during inflammatory events or elevated Zn^2+^ levels that take place during the first two phases of acute wound healing are capable of preventing proteolytic degradation of HRG by plasmin [6,9,28]. Therefore, the examination of the acute wound fluid harboring no proteolytic fragments of HRG infers that the proteolytic breakdown of HRG domains is a result of the upregulation of serine proteases at sites pertaining to chronic wounds (Figure 3B) [35,50,72,81]. This does not rule out HRG as a regulator of the plasminogen system, given that previous studies have demonstrated a tripartite complex with HRG, plasminogen and thrombospondin-1 (TSP-1). HRG can also act individually as a receptor for plasminogen through cell surface tethering to HS regulating PA [9,28,35,54,82,83].

## 6. The Role of HRG in the Innate Immunity and Apoptotic/Necrotic Cell Clearance 

The innate immune response includes an arsenal of complement proteins through three broad pathways—(i) the classical pathway; (ii) mannose-binding lectin (MBL); and (iii) the alternative pathway—that ultimately facilitate the formation of the membrane attack complex (MAC) on microbial pathogens and activation of complement agents that aid in phagocytic engulfment for efficient wound healing. This direct innate immunological response is interlinked through a network of catalytic cascades involving complement proteins that predominantly circulate in the humoral system. This ability to constantly circulate provides a surveillance system to guide innate and adaptive activation through complement receptor binding to sites where ‘danger signals’ normally involve pathogen or cell-induced pro-inflammatory responses. Therefore, the innate system is constantly regulating a balancing act between complement activation and inhibition, that if skewed can promote the risk of uncontrolled cell lysis leading to inflammation and autoimmunity [84,85]. Several studies have shown that HRG may play a pivotal role in bridging many of these innate immune responses by: (i) solubilizing immune complexes; (ii) binding complement components; and (iii) removal of dying cells to aid in tissue repair and prevent immune dysregulation, with Zn^2+^ modulating some of these processes [86,87,88,89,90].

### 6.1. HRG Maintains the Formation of Soluble Immune Complexes

The formation of insoluble immune complexes (IIC) via autoantibodies can occur by binding its cognate antigen or through targeting the fragment crystallizable (Fc) stem of ‘self’ immune complexes (IC). The disease settings involved in the production of autoantibodies include lupus erythematosus, rheumatoid arthritis, infectious mononucleosis and hepatitis C virus infections [87,91]. It is necessary to resolubilize IIC to promote effective removal from circulation, preventing the deposition of precipitating IIC in target tissues and subsequently reducing the onset of immune-complex-mediated tissue injury. The mechanisms in place to counter the formation of IIC include complement components C1q and C3b, with several early reports also linking HRG with this function [87,91,92].

The initial study on HRG involvement on solubilizing IC was by Gorgani et al. [87], with the presence of physiological concentrations of Zn^2+^ also enhancing HRG affinity towards IgG [87]. Interestingly, the enhanced affinity towards immunoglobulin G (IgG) was shown not to be entirely charge dependent, since the affinity was not enhanced in the presence of other divalent metals such as Ni^2+^ and Cu^2+^. The affinities of HRG towards IgG were enhanced by Zn^2+^ > Ni^2+^ > Cu^2+^ (although no K_d_ values were provided) and suggested HRG adopts a unique conformation that enhances its recognition for IgG [87]. Alternatively, these divalent metal co-complexes with HRG might actually correlate to the strength of the divalent metal interaction to HRG (order of affinity), rather than Zn^2+^ inducing a unique conformation to promote binding towards IgG. Nonetheless, the enhanced affinity for IgG in the presence of Zn^2+^ was later identified to be due to the type of light chain, with Zn^2+^-bound HRG exhibiting enhanced affinity towards IgG molecules containing κ-light chains (IgGκ1, IgGκ2, IgGκ3, IgGκ4) [92]. This difference in affinity of HRG for IgG inferred that the solubilisation of IIC might also be dependent on the type of IgG subclasses in the IIC (IgGκ or IgGλ). It is also worth noting that the presence of Zn^2+^ reduced the affinity of HRG towards C1q and IgM [92]. Furthermore, since IgG1κ and IgG2κ constitute 90% of serum IgG, some IgG subclasses/IIC may remain un-cleared from circulation. An explanation behind the un-cleared IIC from the reticuloendothelial system was postulated by Gorgani et al. [92] to prolong the immunological memory [92]. In summary, these data suggest that HRG contributes to maintaining the solubility of circulating IC and protecting against immune complex diseases (ICD) [87,91,92].

#### 6.1.1. Insoluble Immune Complexes and Regulation of Complement System by HRG

The preferential binding of HRG to different IgG subclasses has been demonstrated to negate the formation of IIC and subsequently prevent the onset of ICD, with recent studies investigating the ability of HRG to initiate complement activation [87,91,92]. To address HRG involvement in regulating complement, a study by Manderson et al. [90] showed several complement components tested in vitro and vivo were identified to interact with HRG, including C1q, factor H (FH), C4b-binding protein (C4BP) and IgG (IC or IIC), with C8, C4 and C3 showing weaker affinity [90]. Surprisingly, none of the myriad of complement components that bind HRG had an impact on regulating complement. However, given the complement-activating potential of IIC, the ability for C1q and HRG in the presence of IIC to regulate the complement was further examined. C1q was shown to have greater affinity for IIC and inhibited the complement, while the IIC-HRG complex promoted complement activation [90]. An explanation for these observations was proposed based on how these IIC may interact with C1q and HRG, with C1q promoting IIC by tethering IgG between the Fc and fragment antigen-binding (Fab) interface. In contrast, HRG may inhibit IIC formation by maintaining solubility of individual IC through its N-terminal domains (N1N2) interacting with the antibody Fab region and perturbing Fc/Fc interactions. In the case of the IIC-C1q complex being favored over IIC-HRG complex, it was suggested this was due to HRG binding to the tail region of C1q and allowing unobstructed free access of IgG with the head domain of C1q. Collectively, these complexes (IIC-C1q and IIC-HRG) may also correlate with soluble immune complexes (SICs) being better complement activators than larger SICs, as HRG coordinates with IIC in a monovalent fashion, while C1q bind IgG in a polyvalent manner promoting IIC formation that favors complement inhibition [90].

It is worth mentioning that this study excluded the use of Zn^2+^, due to it being a known complement inhibitor [90,93]. Although since Zn^2+^ is elevated during the initial stages of wound healing (inflammation and proliferation stages), complement activation still perseveres [6,24,27,85,93,94]. Therefore, taking into account the effects of Zn^2+^ on HRG, particularly enhancing HRG affinity towards certain IgG subclasses, it could be assumed that Zn^2+^ may actually favor complement activation [92]. Previously, Zn^2+^ has been shown to reduce HRG affinity for C1q and therefore promote HRG association with certain IgG containing IIC (i.e., IgG2κ) and thus favor formation of HRG-IIC [87,90,92]. This in turn may promote complement activation through maintaining small SIC (HRG-IIC) over inhibiting complement by larger SIC (C1q-IIC) [90].

### 6.2. HRG Potentiates Ingestion of Apoptotic and Necrotic Cell Clearance through Phagocytosis

Apoptosis is a complex, intricate multi-stage process of programmed cell death involving the organized redistribution of sub-cellular compartments, in conjunction with the presentation of unique molecular signatures i.e., ‘find or eat me’ signals that promote subsequent removal by phagocytes to maintain tissue homeostasis [95,96]. While the non-programmed route for cell death, commonly termed necrosis, can be induced via inactive and active mechanisms, these include physical or chemical insult, delayed apoptotic cell clearance, necroptosis and pyroptosis. These inactive and active mechanisms ultimately cause the cellular membrane to become permeable and subsequently enable the release of intracellular contents to trigger an inflammatory response [95]. Therefore, to prevent prolonged inflammatory responses and maintain immunological tolerance, pro-phagocytic agents aid the immediate removal by professional phagocytes to expedite the clearance of damaged cells [95,97]. Recently, HRG was shown to play a role in the removal of dying/dead material by phagocytes, with its activities dependent on a variety of extracellular and intracellular ligands that trigger phagocytosis based upon the stage of cell death [86,88]. In particular, there are observations of HRG interacting with DNA [86], Fc_γ_R [86,88,89], IgG [88,89], HS [98] and enhancing C3b opsonisation (on necrotic cells) [90] to mediate the removal of apoptotic and necrotic cells via macrophages, with the Zn^2+^-modulated processes discussed below [86,89,99].

#### 6.2.1. HRG Potentiates Ingestion of Necrotic Cells by Targeting Heparan Sulfate

Heparan sulfate is a linear polysaccharide that is ubiquitously expressed in mammalian tissues and serves as a reservoir for active and dormant HS-binding proteins. HS contributes to the structural integrity of the ECM on cell surfaces, and when cleaved by its endo-β-glucuronidase (heparanase) can disengage the HS-binding proteins to facilitate immune and inflammatory processes [100]. HRG was shown to facilitate necrotic cell clearance by phagocytes through preferential binding to HS on THP-1 cells (a human macrophage-like monocytic leukemia cell line) [98], as supported by the cleavage of HS leading to reduced binding of HRG^P^ (co-purified IgG with HRG) to THP-1 cells. However, both HRG^PID^ (IgG depleted HRG) and HRG^P^ are still independently capable of promoting phagocytic pathways and it was proposed that each pathway works in unison to facilitate efficient necrotic cell uptake. The study by Poon et al. [98] suggested the necrotic cell recognition region of HRG was localized to the HRR, based on the observation that heparin could inhibit intact HRG but not the N1N2 domain from binding to necrotic cells [56,98]. Therefore, Zn^2+^ is most likely altering the conformational structure of HRG and/or increasing the cationic charge to improve HS binding. As similar mechanisms apply to T-cells, HRG may play a role in guiding multiple cells of the immune system to the wound site for apoptotic, necrotic and pathogen clearance [56,62,98,99].

In summary, these studies illustrate HRG as a pattern-recognition molecule, with similar functionality to other non-homologous proteins such as C1q, C-reactive protein, mannose binding lectin (MBL) and β_2_ glycoprotein I, that guide professional phagocyte interactions with necrotic cells and ultimately promote the formation of the phagocytic synapse. Furthermore, these studies underscore the importance of the source of HRG, since even minute amounts of IgG that co-purify with HRG from plasma are capable of altering the immunological interplay with HRG, thus raising doubts about a number of previous papers on HRG that potentially make incorrect conclusions about the biological activity of HRG [88]. These differences in activity were illustrated by Patel et al. [15], who defined lipid specificity for phosphatidic acid and reduced necrotic cell binding by HRG purified to homogeneity when compared to other standard HRG purification methods that reported broad lipid specificity and enhanced necrotic binding. Regardless, it is clear that IgG plays a crucial role in directing lipid binding; however, other ligands may also interact with HRG to facilitate apoptotic and necrotic cell clearance [15].

## 7. HRG Regulates Angiogenesis during Wound Healing

The blood vessel network that branches to all tissues enables both nutrient and oxygen delivery, whilst also providing a route for immune surveillance [69]. This pre-existing vasculature provides a nucleation point for the growth, maturation, and branching of new blood vessels. This type of vessel formation has been identified as angiogenesis and is a key mechanism of vessel formation that is utilized by the body in wound healing and to counter ischemic tissue [69]. The literature has identified multiple circumstances where HRG could be regulating angiogenic events at sites of wound healing and tumour growth. Considering tumours have been defined as “wounds that do not heal” similar to chronic wounds, HRG may regulate angiogenesis in the tumour microenvironment in addition to sites of classical acute and chronic wounds as discussed below [3,12,19,101].

### 7.1. HRG Potentially Inhibits the Anti-Angiogenic Activity of Thrombospondin 1 and 2 during Acute Wound-Healing Processes

The regulation of angiogenic signals is crucial as the vasculature develops during the proliferative stage of wound healing, in order to provide a source of nourishment for tissue regeneration [20,102]. The TSPs are matricellular glycoproteins, with activities in regulating inflammation, wound healing and cancer progression, with their most noteworthy role illustrated by their anti-angiogenic properties. In particular, two family members, TSP-1 and TSP-2, exhibit potent anti-angiogenic properties mediated through binding to the integral transmembrane scavenger receptor cluster of differentiation 36 (CD36) [83,101]. The presence or absence of these anti-angiogenic molecules is capable of enhancing or delaying vasculature formation, respectively, at sites of tissue injury or tumour growth. This has been observed in inhibition studies of TSP-1 facilitating wound closure whereas overexpression of TSP-1 was shown to perturb wound healing [83]. Other studies in mice *TSP-2*^-/-^ mice, however, have demonstrated an increased wound-healing proficiency of excisional wounds [101]. Therefore, due to the expression patterns of TSP-1 and TSP-2 into the wound bed and HRG capacity to subdue their anti-angiogenic activities, it is a possibility that HRG could be regulating the timing of these angiogenic events during wound healing [83,101].

The ligand-binding domain of TSP-1 contains properdin-like or thrombospondin structural repeats (TSR) that harbor anti-angiogenic properties. Structural analysis of the TSP-1 TSR domain has suggested that its anti-angiogenic action is mediated via the cationic charge interface of a groove-like structure termed *C*D36 LIMPII Emp *s*tructural homology domain-1 (CLESH-1) motif to accommodate ligand interactions with molecules such as CD36 and HRG [101]. CLESH-1 motifs were also identified in proteins outside the CD36 family such as lysosomal integral membrane protein II, human immunodeficiency virus (HIV) envelope glycoprotein 120 and HRG. In order to establish how HRG in an in vivo setting is regulating the anti-angiogenic properties of TSP-1 and TSP-2, a cornea model from mice was used to investigate vasculature formation. It was clear that the presence of HRG impeded the TSP-1 and -2 anti-angiogenic activity, which suggests that HRG acts as a soluble decoy receptor by interacting with TSP-1 and -2 via its CLESH-1-binding domain (amino acids 446-507) to inhibit TSP-1 and -2 binding to CD36 and therefore promote angiogenesis [83,101]. These interactions are further supported by recent studies highlighting the significance of peptide mimetics derived from the TSR domain of proteins such as TSP-1 in reducing angiogenesis of tumours by inhibition of CLESH-1-containing proteins such as HRG or CD36 [103].

**Figure 4:**
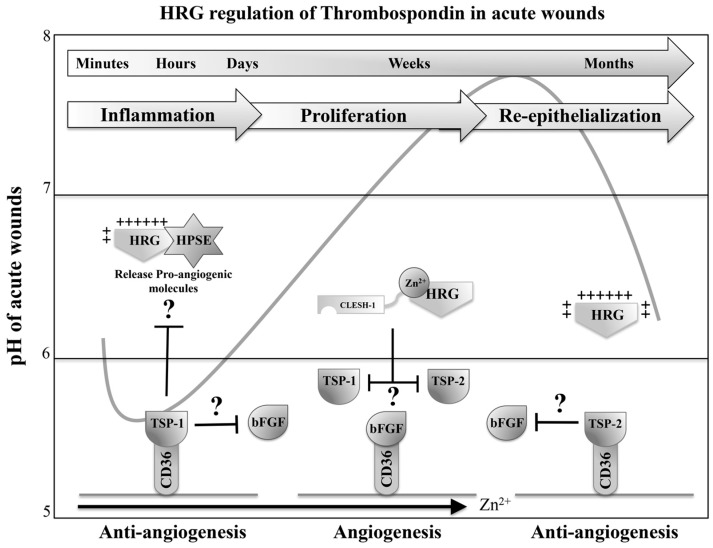
Predicted representation of thrombospondin (TSP) regulation by HRG during acute wound healing. HRG has been shown to enhance the enzymatic activity of heparanase (HPSE) under acidic conditions and therefore may work in unison in aiding cell migratory process as the extracellular matrix (ECM) constituent heparan sulfate (HS) is cleaved, while allowing TSP-1 to prevent angiogenic stimulation from the subsequent release of pro-angiogenic moieties such as basic fibroblast growth factor (bFGF) [6,12,83,101,104]. The differential expression patterns of TSP-1 and TSP-2 may allow HRG to guide angiogenic activation that flank each side of the proliferative stage of acute wound healing [101]. Figure adapted from Schneider et al. [6], Li et al. [105] and Simantov et al. [83]. CD36: cluster of differentiation 36.

This angiogenic switch that is regulated by the CD36/TSR pathway and CLESH-1-containing proteins is likely controlled by the expression patterns of TSP-1 and TSP-2 [101]. Quantitative mRNA analysis showed that TSP-1 expression is elevated during the first 24–72 h and TSP-2 at 3–10 days after wounding, illustrating that there may be anti-angiogenic regulation occurring at different phases of wound healing with HRG potentially playing a pivotal role in regulating the timing of these angiogenic events [83,101]. Therefore, since many of the previously established HRG ligands are pH sensitive, it is reasonable to suggest that these interactions with TSP-1 and TSP-2 may also be subject to pH restrictions (Figure 4). It is worth noting that these TSP interactions have not been examined in the presence of Zn^2+^ and hence it cannot be excluded that the HRR may bind Zn^2+^ during the proliferative stage of the wound-healing process to disrupt the interactions with TSP-1 and TSP-2. However, since the CLESH-1-binding domain of HRG is localized to the C-terminal domain, it may remain sufficiently distant from the HRR in order to withhold a localized negative charge that may otherwise be masked by the positive charge induced through Zn^2+^ binding [4,83,101].

### 7.2. Chronic Wound Environment and the Potential Role of HRG

The chronic wound environment is characteristically associated with impaired vasculature development during the proliferation stage of wound healing and therefore the healing process tends to stagnate in the inflammatory stage [106]. The reduction in vascular density has been attributed to the expression of MMP and their inhibitors (TIMPs). In particular it has been demonstrated that MMP are upregulated in chronic leg ulcers such as MMP-1, -2, -8, and -9, while TIMPs are downregulated [107]. The excessive presence of these matrix-degrading enzymes is through constitutive secretion from neutrophils, macrophages and leukocytes that persist in the inflammatory response. Furthermore, their sustained activity contributes to impaired vascular development and eventually leads to tissue disruption [107,108]. The non-healing phenotype of chronic wounds can result in prolonged exposure to the environment that typically increases susceptibility to microbial infiltration within the necrotic debris and wound fluid [106,107]. In addition, the chronic wound environment is Zn^2+^ deficient with declining physiological concentrations of 20 μM to ≤ 9 μM and harbors an alkaline environment. These environmental attributes have been shown to facilitate excessive protease activity that in combination with previous factors involving impaired vascular development and a Zn^2+^-deficient environment, contributes to impaired wound healing [6,11]. Therefore, since chronic wounds do not meet the acidic or Zn^2+^-rich prerequisite for peptides containing the HRR of HRG to exert its anti-angiogenic or antimicrobial activities, it is likely that the activities of HRG are differentially regulated between the chronic wound and tumour setting.

In the case of the proteolytic breakdown of HRG as previously observed in chronic wound fluids by Rydengård et al. [35], the environment preferentially facilitates excessive proteolytic activity of plasmin, elastase and cathepsin-G that appear to be prime candidates for proteolytically generating potentially active domains of HRG [35,50]. Interestingly, in the case of plasmin-cleaved HRG or the presence of peptides containing the HRR, it does not appear to contribute to the progression of chronic wounds. However, there might be molecular features within the HRR or protease-cleaved HRG that may assist in restoring acute wound-healing processes. In particular the ability of plasmin-cleaved HRG to reduce plasmin turnover via sequestering plasminogen to GAG surfaces, may be an attempt to suppress excessive proteolytic activity [28]. This suppression may extend to other proteases, as dysregulation of proteases is a key feature of chronic wounds [50]. In addition, the ability of plasmin-cleaved HRG to enhance necrotic cell clearance is also an important mechanism prior to wound closure [11,28]. Furthermore, the known anti-angiogenic activities of the HRR should not contribute to chronic wound progression, given Zn^2+^ is a prerequisite [12]. Collectively, this infers that the cleavage of HRG and the subsequent release of potentially active domains may be a response mechanism to chronic wounds that have diverged from acute wound-healing mechanisms (Figure 3B).

#### 7.2.1. HRG Anti-Angiogenic Peptide Suppresses Tumour Growth

The stromal components (fibrin, fibronectin, collagen type I and III) between the tumour and that of healing wounds are essentially identical, with the main feature that distinguishes tumours being the constitutive expression of these stromal components [19,109]. The concept of tumours mimicking that of chronic wounds as previously described as “wounds that do not heal” does not extend to the microenvironment [12,19]. The tumour environment has been shown to be quite acidic and have elevated concentrations of Zn^2+^ through excessive platelet activation, which is opposite to what is observed in chronic wounds [6,10,11,12,26]. Despite these differences, the generation of peptides that contain the HRR can still be proteolytically released from HRG in both environments, with known factors of low pH and Zn^2+^ present in the tumourigenic environment not sufficient to impede the proteolytic degradation of HRG [10,12,28,35]. Therefore, it is likely that there are different types of proteases in the chronic or tumour environment capable of differentially regulating the proteolytic breakdown of HRG [50,110]. The peptides derived from the HRR have been demonstrated in multiple studies to have anti-angiogenic properties compared to the parent protein, with protonation through an acidic environment or the presence of Zn^2+^ critical to exert this type of activity [12,64,75,77,79]. Therefore, in the case of the tumourigenic environment and the presence of this anti-angiogenic peptide (HRGP330) observed in the plasma of healthy and cancer patients, it can be deduced that HRG is involved in suppressing tumour progression [12].

The evidence supporting that different proteases likely regulate HRG between the chronic and tumour microenvironment, is based on the following papers by Thulin et al. [12] and Rydengård et al. [35]. The detection of the fragment containing the anti-angiogenic peptide (HRGP330) in the plasma of healthy and cancer patients was confirmed by Western blot under non-reducing conditions at ~30 kDa and by mass spectrometry [12]. This size is slightly smaller than the proteolytic fragment of HRG observed under non-reducing conditions in the chronic wound fluid fraction at 36 kDa [35]. Interestingly, the study by Thulin et al. [12] attempted to identify the source of the protease that was acting on HRG from thrombin-activated platelets, although to no avail. This suggests other proteases may be required at sites of the tumour microenvironment that are not proteolytically hindered by bound Zn^2+^ with non-cleaved HRG or protonated HRG resulting from the acidic microenvironment of developing tumours [10,28]. Therefore, in comparison to the intact HRG from acute wounds, the fragments containing the HRR in the chronic wound and the tumour microenvironment that are commonly described as “wounds that do not heal,” strongly suggests that the release of peptides derived from HRG HRR may be a by-product of impaired wound healing [12,19,35].

## 8. Concluding Remarks

Our understanding of Zn^2+^-regulated biological processes has increased in the past decade, including the properties that are essential in facilitating structural or catalytic activities of proteins. The observed Zn^2+^ binding to histidine-rich glycoprotein appears to regulate its function at sites pertaining to wound healing and is mediated through the pH and Zn^2+^-sensing ability of the histidine-rich region. It is evident that the large number of proposed HRG ligands cannot be accommodated simultaneously, therefore it is plausible the HRR may finely tune individual interactions by modifying the native or cleaved form of HRG by altering its conformational structure through the charged state of histidine, induced by acidic pH or binding to Zn^2+^ (Table 1). The HRR may therefore act as the regulatory module throughout each of the wound-healing stages and hence restrict the type of ligand interactions that can take place to facilitate biological processes that contribute to efficient tissue regeneration.

## Figures and Tables

**Table 1:** Histidine-rich glycoprotein ligand interactions that can facilitate immunological or vascular processes, with the tick (✔) denoting the type of physiological setting wherein the interaction can take place; the cross (✖) denotes which condition was tested and no interaction was observed; no change (N/C) denotes that Zn^2+^ had no impact on the interaction; and not determined (N/D) denotes the interaction has not been tested.

	HRG Ligand Interactions					
		**Zn^2+^ regulated interactions**		**pH changes**		**References**
	**HRG Ligands**	Promoted by Zn^2+^	Inhibited by Zn^2+^/or reduced affinity	Acidic (pH < 7.4)	pH ≥ 7.4	
**Immunological Processes**	Heparin	✔		✔	✔	[55,65,66]
	Heparan Sulfate	✔		✔	✔	[56,64]
	C1q		✔	N/D	✔	[87,90]
	Microbes	✔		✔	✔	[35,49,67]
	IgG1κ	✔		N/D	✔	[92]
	IgGλ		✔	N/D	✔	[92]
	ATP Synthase	N/D		N/D	✔	[111]
	Phosphatidic acid	N/D		N/D	✔	[15]
	Heparanase	N/C		✔	✔	[104]
						
**Vasculature Processes**	Vasculostatin	N/D		N/D	✔	[112]
	Thrombospondin-1	N/D		N/D	✔	[83]
	Thrombospondin-2	N/D		N/D	✔	[101]
	Plasminogen	N/C		✔	✔	[28,54]
	Plasmin		✔	✖	✔	[28]
	Fibrinogen	✔		✖	✖	[53,61]
	Fibrin	✔		✖	✖	[53,61]
	Tropomyosin	✔		N/D	✖	[77]
	FXIIa	✔		N/D	✔	[14]
	α_v_β_3_	✔		N/D	✔	[79]
	Heparanase	N/C		✔	✔	[104]
	Heparin	✔		✔	✔	[55,65,66]
	DNA/RNA	N/D		N/D	✔	[58,86]
